# Grip-force modulation in human-to-human object handovers: effects of sensory and kinematic manipulations

**DOI:** 10.1038/s41598-020-79129-w

**Published:** 2020-12-24

**Authors:** Falko R. Döhring, Hermann Müller, Michael Joch

**Affiliations:** 1 Neuromotor Behavior Laboratory, Institute of Sport Science, Justus-Liebig-University, Kugelberg 62, 35394 Giessen, Germany; 2 Center for Mind, Brain and Behavior-CMBB, Universities Marburg and Giessen, Marburg, Germany

**Keywords:** Motor control, Human behaviour, Sensorimotor processing

## Abstract

From a motor control perspective, human-to-human object handovers can be described as coordinated joint-actions transferring the power over an object from a passer to a receiver. Although, human-to-human handovers are very reliable in terms of success, it is unclear how both actors plan and execute their actions independently while taking into account the partners behaviour. Here, we measured grip-forces of passer and receiver while handing over an object. In order to study mutual interaction in human-to-human handovers, we measured how changes in relevant features (sensory information available to the passer and receiver’s reaching velocity) in one partner affect grip-force profiles not only at the manipulated side but also at the partner’s side. The data reveals strong effects of sensory manipulations on time-related (duration and release delay) and dynamometric measures (force rates). Variation of reaching velocities had the largest impact on the receiver’s force rates. Furthermore, there are first indications that the vertical object movement is used as an implicit cue to signal the start of the handover in situations where vision is restricted.

## Introduction

When we imagine passing an object like a glass of water to another person in daily live, we might think that there is nothing special to it, it just works. Humans are so well trained on handing over objects, that those handovers are successful with all kinds of objects like tools, keys or ropes. Irrespective of the exact properties of an object and the experience and skilfulness of person we are interacting with, human-to-human handovers have an astonishing rate of success, while being considered highly automated at the same time^1^. One crucial element in this particular skill is the human capability to adapt grip-forces to a wide range of different tasks and objects^2,3^. One would expect that such a seemingly easy action is already well understood and can easily be modelled. However, the struggles in robotics trying to implement human-to-robot or robot-to-human handovers highlight the lack of knowledge about the control mechanisms underlying human-to-human handovers.

Human-to-human handovers involve two separate control systems (two persons), each of them having to account for each other not only during the handover itself, but also when planning its individual movement. The person passing an object is typically called *the passer*. On the other side, the person receiving an object is called *the receiver*. The action itself can be roughly divided into three phases^4,5^: (1) the *transport phase passer*, (2) the *handover*, and (3) the *transport phase receiver* (see Fig. 5b). In the first phase, the passer carries the complete load of the object alone and presents the object to the receiver (either by holding it statically or by moving it towards the receiver). During that phase, both persons already plan their own motor actions for the ensuing *handover* phase, taking into account the predicted behaviour of the partner. In the *handover* phase, both persons are interacting with the object simultaneously. The crucial part is that both persons have to synchronize their rates of force increase (receiver) and force reduction (passer) in order to assure a smooth transfer. This is also the characteristic phase that distinguishes handovers from other joint-actions, e.g. throwing an object to a partner, joint pickup-and-place tasks^6^, or when the object is picked up from the table by the receiver^7^. The third and last phase begins when the passer has fully released the object, and the receiver holds the object alone.

Although there is not much research on the underlying control mechanism of human-to-human handovers yet, existing studies focused primarily on the *handover* phase.

A study by Mason and MacKenzie^5^ verifies that both partners synchronize their rate of change in grip-force applied to the object during the handover. They could also show that a typical object handover has an average duration of about 500 ms. They also demonstrate that the temporal characteristics of the handover phase are mostly unaffected by the type of passer’s transport (static: passer holds the object in one place during phase one vs. dynamic: passer moves the object towards the receiver in phase one). This is true although the horizontal object displacement was affected by the type of transport. This suggests, that not all three phases of the handover are pre-programmed. At least the *handover* phase seems to be controlled separately, involving feedback-based control mechanisms to account for unpredictable behaviours of the partner, thus assuring a save transfer of the object^5,8^. This feedback might rely on different sensory modalities. A recent study by Controzzi et al.^8^ could show that the modulation of grip-forces during the handover phase depends on the availability of visual information to the passer. They found that handovers become more reactive when the passer is blindfolded but in return, the passers reduced their grip-forces with a higher rate in the blind condition compared to a normal condition where vision was not restricted. The increased force reduction rate is assumed to be a compensatory mechanism to account for a delayed onset of the release. They could also show that the handover duration depends on the velocity of the receiver’s hand when reaching for the object, which can be interpreted as indication for the relevance of visual input not just for feedback control but also for pre-programming of release.

The majority of reported effects so far are related to the timing of events during a handover, emphasizing the notion that exact temporal control is a very crucial factor for successful handovers. However, requirements for temporal acuity are largely different for different manners of handover. In the study of Controzzi et al.^8^, subjects had to pass a light object in a horizontal orientation. Due to the weight of the object, receivers are able to build up the necessary grip-force to hold the object relatively quickly. Thus, the actual effect of the temporal precision and its consequences could be reduced when handing over light objects. Furthermore, when handing over objects in a horizontal orientation, both actors must account for the torques. An issue arising from torques when grasping an object was descripted by Schneider et al.^9^. They showed that participants are unable to reliably predict torques when planning a grasping-movement and cannot compensate for errors in subsequent trials. Hence, the noise introduced by unreliable torque prediction in horizontal object handovers makes it harder to investigate temporal characteristics of the load-force transfer from passer to receiver. In a vertical object orientation, the influence of torques are reduced and grip-forces are closer to the load-force of the object. In consequence, measured grip-forces can be more directly connected to the transfer of the object’s load force and temporal aspects of the handover can be investigated more reliably.

Hence, the first aim of the current study is to test whether the availability of visual and tactile information during handover affects control of grip-forces in a similar fashion when a relatively heavy object is transferred in a vertical orientation (Fig. 5a). Similar to previous studies, the two independent variables are the sensory status of the passer and the reaching velocity of the receiver (Fig. 5c). The questions we seek to address with this first aim are as follows.

- How does the passer modulate his/her grip-force behaviour when having restricted sensory information about the receiver’s reaching movement and the interaction itself?
- How does the passer behave when the receiver approaches the object with different reaching velocities?
  In addition to the manipulation of visual information, we reduce tactile information of the passer to test whether feedback-based control during the handover phase is more driven by visual or tactile information.
  Additional to these research questions that relatively directly build on previous research, our study pursued a second, more exploratory aim. Existing studies mainly looked at effects on the behaviour of the passer while experimental manipulations were also applied to the receiver. In order to have a closer look on the interaction, we also look at behavioural changes of the receiver when the sensory manipulation is applied to the passer.
- Does the receiver account for sensory restrictions of the passer?

As stated earlier, handovers are highly practiced. The extensive practice we go through in our life may allow us to predict the behaviour of our partner based on our knowledge about his/her situation^10,11^. If feasible, we could anticipatorily adjust our own control based on the current status of our partner. Alternatively, our adjustments may also be purely reactive to sensed changes in the behaviour of the other person.

In order to differentiate between predictive (pre-programmed) and feedback-based control within the *handover* phase, it is necessary to define two sub phases that divide the *handover* phase into a (necessarily) predictively controlled and a (potentially) feedback-controlled phase (see Fig. 5b). The predictive control phase includes pre-programmed grip-force modulation that cannot be affected by sensory information from the interaction due to physiological feedback processing latencies. If we assume that tactile sensory feedback about the object can be processed within the first 80–100 ms^12^, any changes in the first approx. 100 ms after receivers contact are necessarily pre-planned. Accordingly, a full loop of sensory modulated information processing from passer to receiver and back takes at least 200 ms. It takes the receiver 100 ms to react to any changes of grip-force by the passer. At least another 100 ms will elapse before the passer detects receiver’s reaction and will be able to react to it by him-/herself. Thus, the earliest time where we can expect a complete back and forth interaction of passer’s and receiver’s grip-force modulation is at about 200 ms after initial contact of the receiver. Any changes in grip-force in the rest of the handover phase, are possibly modulated by sensory feedback about partner’s behaviour.

## Results

### Control of experimental manipulations

**Figure 1:**
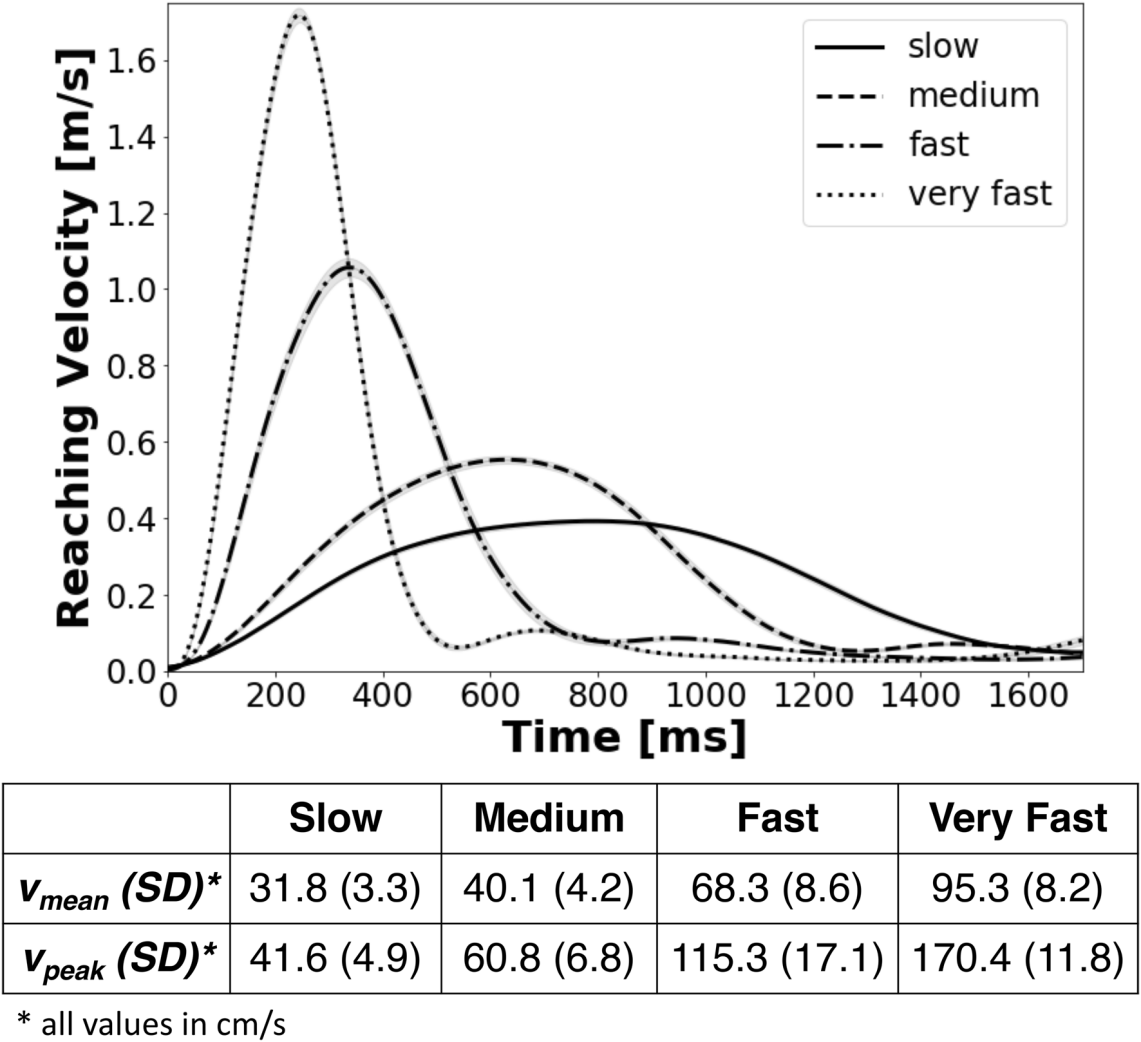
Velocity profiles of the receiver’s reaching velocity. Confidence bands were generated using a point-by-point bootstrap approach by Joch et al.^16^. The table includes measured mean and peak velocities for all four velocity variations.

The reaching velocity of the receiver varied across the experiment in four conditions (slow, medium, fast and very fast). The actually produced average reaching velocities are depicted in Fig. 1. This confirms clear differences between the velocity profiles of all four conditions. Similar effects were found in the analysis of peak velocities. Thus, all four velocity variations are different enough to investigate effects resulting from differences in the receiver’s reaching velocity.

### Sensory status of the passer

**Figure 2:**
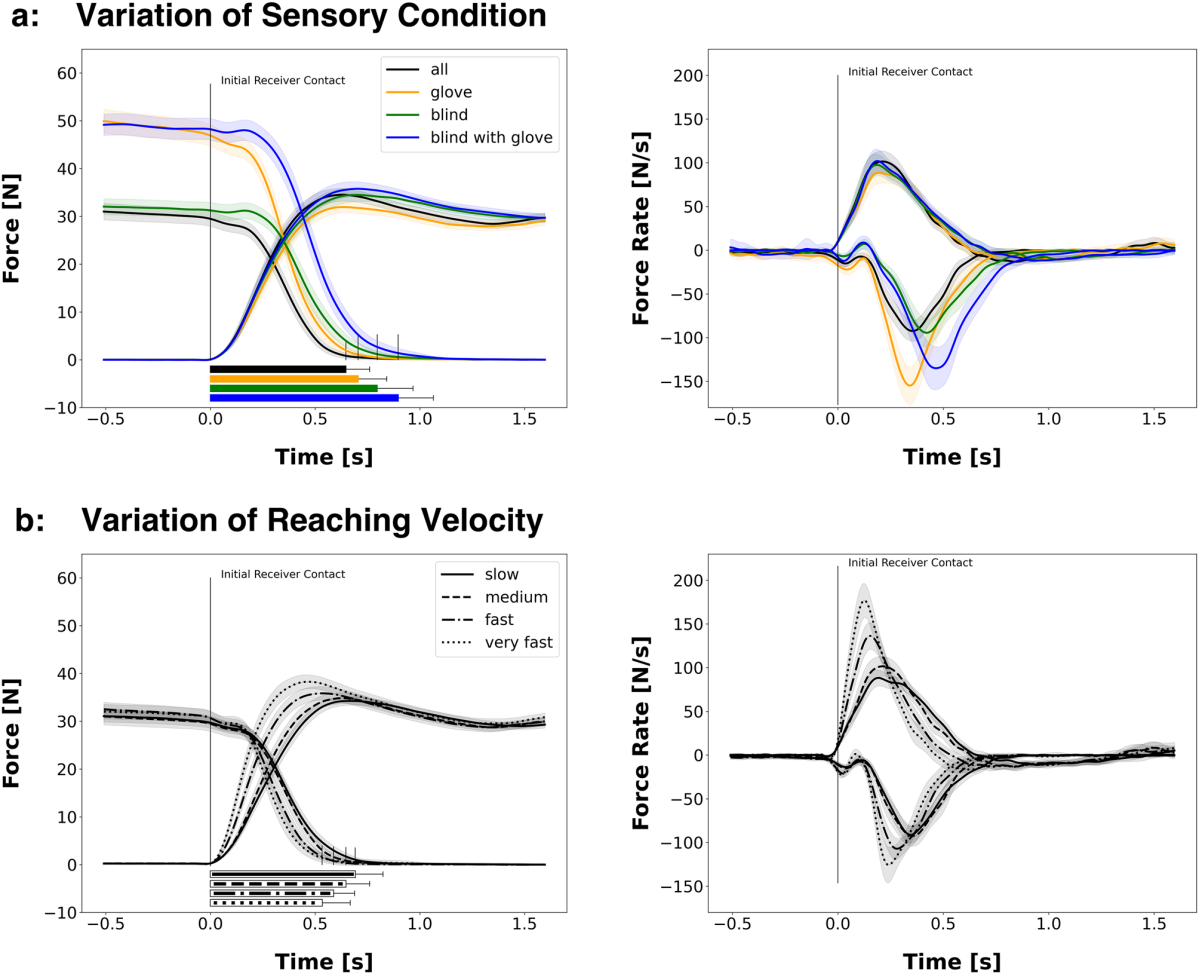
(**a**) Force profiles showing the effect of sensory manipulation of the passer during handover (velocity condition = *medium*). Horizontal bars below illustrate the average handover duration of each condition. The right diagram shows the force rates of the passer (negative rates) and receiver (positive rates). (**b**): Force profiles of passer and receiver showing the effect of the variation of reaching velocity (sensory condition = *all*). The right diagram shows the corresponding force profiles.

In general, grip-forces of the passer are higher in conditions were the glove was used to reduce tactile information (Fig. 2a left, orange and blue; Fig. 4d). More specifically, whereas the restriction of visual signals lead to a grip-force increase of only 1 N, wearing a glove increased the passer’s grip-force by 16.3 N in the glove condition and by 18.6 N when both sensory modalities were manipulated. This can be explained by the reduced friction of the glove^13^, which induces an increased uncertainty and, therefore, higher safety margins of the tactile signals^14^. In order to prevent the object from slipping, the reduced friction has to be compensated by a higher grip-force. Interestingly, the receiver uses less grip-force in the condition, were the passer is wearing the glove but has visual information compared to all other conditions (*F*(3,21) = 5.4, *p* = 0.002, *BF*_*10*_ > 100; all PostHoc comparisons involving the glove condition show significant differences). However, receiver grip-force modulation is unaffected in all other variations of the passer’s sensory status (PostHoc *all* vs. *blind*: *BF*_*10*_ = 0.18; *all* vs. *both*: *BF*_*10*_ = 0.13; *blind* vs. *both*: *BF*_*10*_ = 0.34).

The time span between the receiver’s initial contact with the object and the passer’s start to release grip-force (i.e. *Onset-Delay*) is modulated by the sensory information available to the passer (*F*(3,21) = 59.5, *p* < 0.001, *BF*_*10*_ > 100). In detail, the Onset-Delay becomes larger when sensory information of the passer is manipulated (PostHoc *all* vs. *blind*: *BF*_*10*_ > 100; *all* vs. *glove*: *BF*_*10*_ > 100). Although all comparisons involving the baseline condition (*all*) show statistically significant differences, mean differences show that removing visual information has a larger impact on the Onset-Delay than the manipulation of tactile signal by the glove (mean diff. *all*-*blind* = 50 ms; mean diff. all-glove = 22 ms; Fig. 4e). This result indicates that the passer’s force reduction becomes increasingly reactive the more prediction-related sensory signals are restricted. Hence, the data suggests a higher predictive value for the visual signals than for the tactile signals.

Sensory manipulations also have a major impact on the passer’s average force reduction (Fig. 4a; *F*(1.80,21) = 52.3, *p* < 0.001, *BF*_*10*_ > 100). Wearing a glove leads to a highly significant increase of force reduction from 61.6 N/s in the baseline condition to 89.5 N/s in the glove condition (PostHoc: *t*(21) = − 6.8, *p* < 0.001, *d* = − 1.5, *BF*_*10*_ > 100). In contrast to the glove condition, the removal of visual information leads to a slight decrease of the passer’s force release rates (43.8 N/s in the blind condition; PostHoc *all* vs. *blind*: *t*(21) = 3.9, *p* = 0.001, *d* = 0.85, *BF*_*10*_ > 100).

Handovers become increasingly long when restricting sensory information to the passer (Fig. 4c; *F*(3,21) = 69.8, *p* < 0.001, *BF*_*10*_ > 100). In detail, removing visual information has a higher impact on the handover duration (*all* vs. *blind* condition: *BF*_*10*_ > 100) compared to the damping of tactile signal by the glove (*all* vs. *glove* condition: *BF*_*10*_ = 47). The prolonging of handover duration when restricting visual signals can be explained by the increase of the Onset-Delay. Concretely, in the medium velocity condition, the baseline handover duration was 643 ms ± 71 ms. The glove extended the duration to 712 ms ± 112 ms, and the removal of visual signals made handovers even longer (796 ms ± 141 ms). The longest handover durations were measured in the condition where visual and tactile signals were disrupted (885 ms ± 132 ms). The same systematic can be observed in all other velocity conditions.

**Figure 3:**
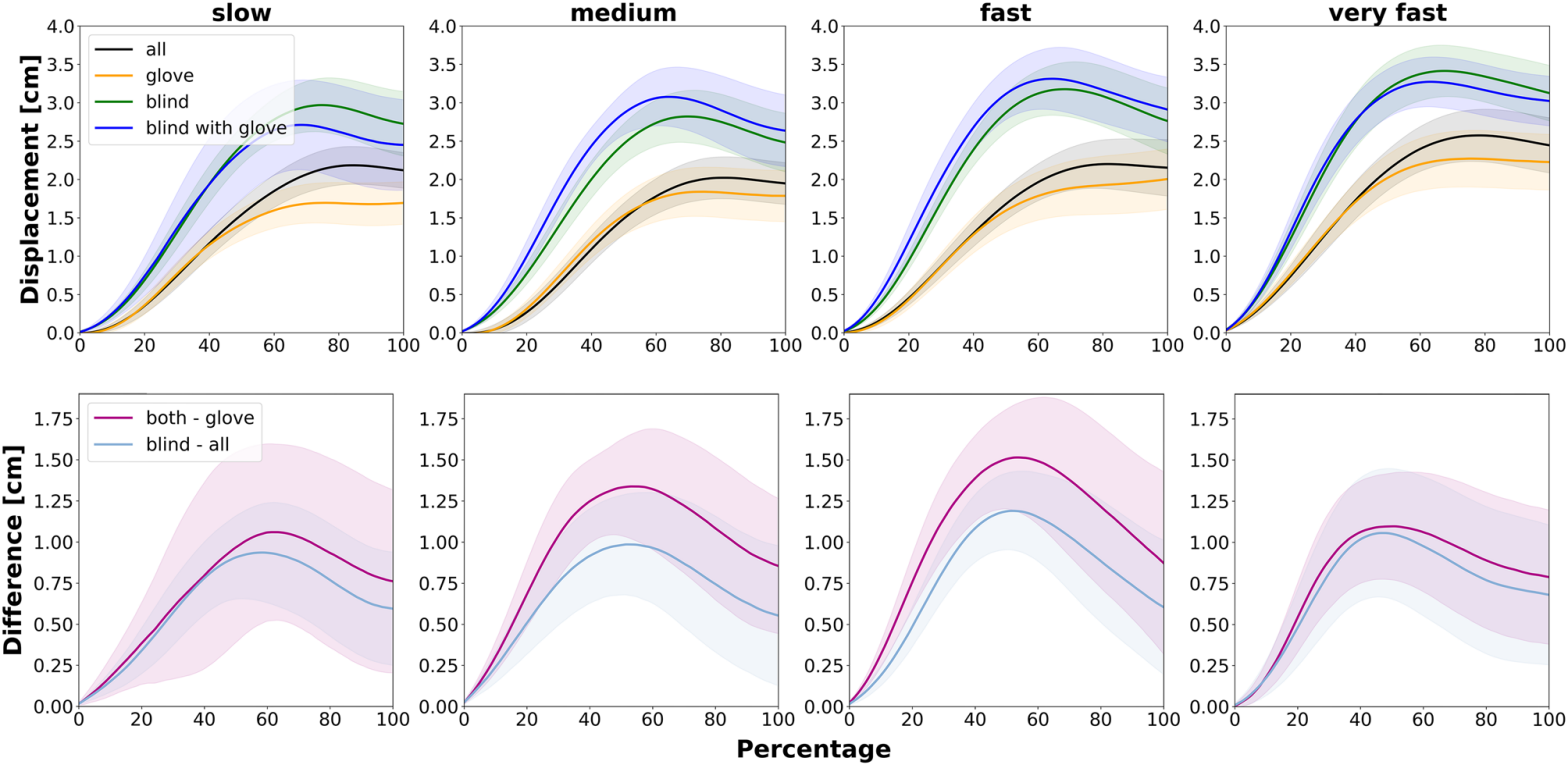
Kinematic profiles showing the object movement in the vertical direction during the handover phase (upper row). The profile begins at the moment of initial receiver contact (0%) and ends with the last contact of the passer (100%). Height values were baseline corrected to the object height shortly before the receiver’s initial contact with the object. The profiles in the lower row show the isolated effects the visual restriction. Error-bands show 95% confidence interval by PBRT algorithm.

In an exploratory analysis of the object movement during the handover phase (i.e. passer and receiver simultaneously on the object), there was significantly more vertical object displacement when the passer had no access to visual information (Fig. 3, *p* < 0.05 indicated by PBRT confidence bands). Both difference curves (*blind*—*all*; *blind with glove*—*glove*) differed significantly from zero indicating a higher vertical object displacement in the conditions *blind* and *blind with glove*. Interestingly, the variation of the receiver’s reaching velocity had no further impact on the vertical object displacement (*F*(3,21) = 1.4, *p* = 0.26, *BF*_*10*_ = 0.17).

### Variation of receiver’s reaching velocity

The passer’s grip-force when statically holding the object just before the handover phase is only marginally influenced by the receiver’s reaching velocity. Although the repeated measures ANOVA shows a highly significant effect for velocity factor (*F*(3,21) = 20.1, *p* < 0.001), the passer’s force only changes from 38.5 N in the slow to 40.7 N in the very fast condition. The Bayesian analysis supports the conclusion, that the factor velocity has no practical relevance (comparison of null model to factor velocity *BF*_*10*_ = 0.03).

**Figure 4:**
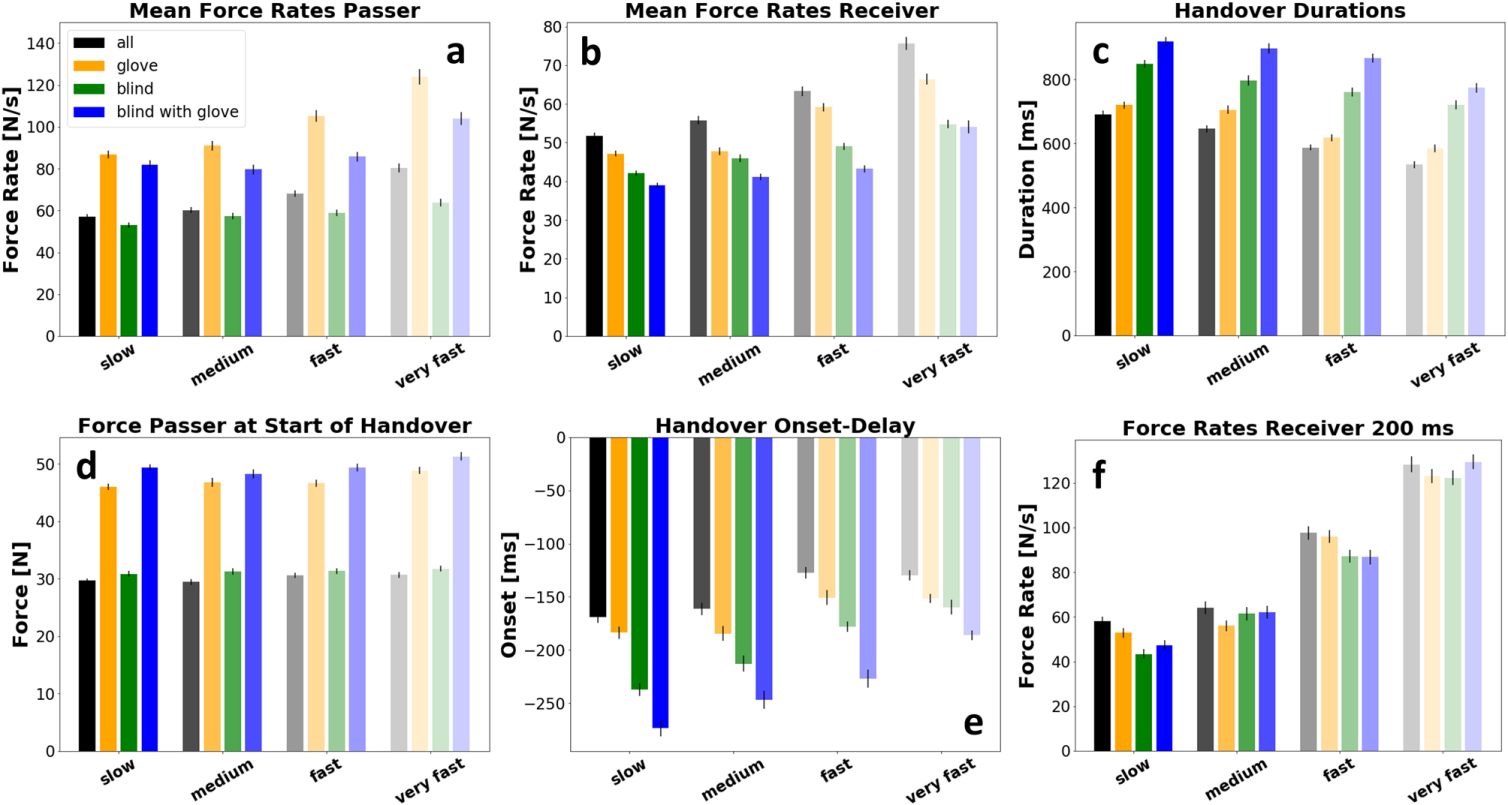
Bar plot for each analysed parameter showing effects from sensory manipulations (colours) and reaching velocity (bar groups). Error bars show standard error of the mean.

However, the variation of reaching velocity has a large effect on the force rates of the receiver (see Fig. 4b) when building up force on the object (*F(1.5,21)* = *91.9, p* < *0.001, BF*_*10*_ > *100*). More specifically, the force rates are gradually inclining when also increasing the reaching velocity from slow (i.e. 32 cm/s) to very fast (i.e. 95 cm/s). The upregulation of force rates in fast conditions also leads to a stronger grip-force overshoot during handover (Fig. 2b). When slowing down the reaching movement, this overshoot becomes smaller. Interestingly passers react to the change of receiver’s force rates by adapting their force rates to those of the receiver (*F*(1.6,21) = 61.3, *p* < 0.001, *BF*_*10*_ > 100). As a consequence of the increased grip-force rates, handover durations are also modulated by reaching velocity (*F*(2.1,21) = 54.8, *p* < 0.001, *BF*_*10*_ > 100). When looking at the *Onset-Delay*, there is a significant interaction effect (*sensory condition* × *reaching velocity*: *F*(3,21) = 3.2, *p* = 0.001, *BF*_*10*_ > 100). Concretely, there is a tendency for a reduction of the delay in fast and very fast conditions. However, this reduction is more pronounced in conditions where the passer had no visual information about the receiver’s reaching velocity (see Fig. 4e).

### Receiver’s behaviour in the predictive control phase

The predictive control phase was defined as the first 200 ms after the initial contact of the receiver with the object. In this phase, pre-planned grip-force modulation can be assumed. The data reveals a strong effect of the reaching velocity variation (Fig. 4f). This effect is most likely the connection between reaching velocity and grasping-speed. This idea is empirically supported, by a significant correlation between the reaching velocity and the mean force rate in the first 200 ms (r(2754) = 0.62, p < 0.001). The sensory manipulation lead to significant differences in the repeated measurement ANOVA (*F*(2.27,21) = 3.1, *p* = 0.05), a look at the mean differences and the Bayesian analysis (*BF*_*10*_ = 0.04) show that this effect is practically irrelevant.

## Discussion

In this study we tested the influence of visual and tactile signals of the passer on the grip-force modulation when handing over an object to a partner. Participants were asked to hand over an object to the experimenter, who acted as the receiver. We measured grip-forces of both persons and varied the availability of visual and tactile signals available to the passer and the reaching velocity of the receiver. In contrast to previous studies by Controzzi et al.^8^ and by Mason and MacKenzie^5^, we changed the handover task so that the interaction between the persons becomes time critical. Handovers in this study had to be executed with a heavier object (1.8 kg) orientated vertically. In addition to dynamometric parameters, hand and object positions were recorded using a motion capture system. The aims of this study were formulated in three research questions.

### How does the passer modulate his/her grip-force behaviour when having restricted sensory information about the receiver’s reaching movement and the interaction itself?

The data shows that two different compensating mechanisms can be distinguished depending on the modality that is manipulated. Removing visual information reliably leads to delayed start of grip-force reduction by the passer, whereas the rate of force decrease is kept unchanged (compared to normal condition). The increased of onset delay is consistent with the findings of Controzzi et al.^8^ and can be explained by fact that the timing of force release onset cannot be anticipated by the use of visual information provided by the receiver’s reaching movement. However, opposed to Controzzi’s study, we do not find any differences in force reduction rates when releasing the object. The increased onset delay also transfers to a prolongation of the duration of the handover phase. The handover duration is prolonged by the duration of the onset delay.

In conditions where the passer was wearing a glove, grip-forces of the passer are generally higher than without gloves. In addition, passers increase their force reduction rates when wearing a glove which leads to a faster object release. Although force rates of the passer increase, the overall handover durations are prolonged compared to the normal condition. These results extend the existing knowledge coming from a study by Endo et al.^15^, where the receiver was wearing a glove during the object handover. In their study, a 10 ms increase in handover durations was found even though the passer was not affected by the manipulation. The most significant difference between the current findings and the results of Endo et al. is that grip-forces of the person wearing the glove are significantly higher in our data whereas they remain unchanged in the data reported in the Endo study. It is assumed that the glove used by Endo was thin and did not affect the friction coefficient of the acting hand as opposed to the thick wool glove that was used in this experiment. Furthermore, in the condition where visual and tactile signals were simultaneously restricted, both effects add up. Concretely, the force rates are comparable to the glove condition and the onset delay is similar as observed in the condition were only vision was restricted.

Differences to existing studies^8^ can be attributed to the different handover setup, i.e. heavier and vertically oriented object. These two factors potentially have a major effect on the grip-force modulation because passing light objects in a horizontal orientation are more robust to suboptimal synchronization between passer and receiver. For instance, when objects are passed in a horizontal orientation, the beginning of the force reduction does not have to be precisely timed because the consequence would only be a downward tilt at the distal end of the object. In the vertical configuration, releasing the object too early directly leads to a downward acceleration and in consequence to a drop of the object. Hence, relatively high onset delays in this study can be attributed to increased safety thresholds of the passer.

### How does the passer behave when the receiver approaches the object with different reaching velocities?

In general, handover durations become shorter when speeding up the receiver’s reaching velocity. The factor that primarily contributes to this reduction is the increase of force rates in both persons. The data also shows that the grip-force, which is used to statically hold the object, is not influenced by the variation of reaching velocities. This might be an effect of the weight of the object, since the forces introduced by the collision might be minor compared to the grip-force that is needed to hold the object itself in a stable manner. As mentioned above, passer’s force reduction rate is related to reaching velocity of the receiver. This effect is not surprising since the passer’s motor system can plan the force reduction based on information of the receiver’s reaching movement. Thus, when perceiving high reaching velocities, passers might anticipate a rapid force increase of the receiver when grasping the object. The prediction of force rates of the handover partner is an essential input for own motor planning. If a high force increase of the receiver is not answered by a high force reduction of the passer, the force rate synchronization is unbalanced, which would possibly lead to an unnatural handover feeling.

### Does the receiver account for sensory restrictions of the passer?

If the receiver’s movement planning is influenced by the manipulations applied to the passer, effects should show in the pre-planned grip-force modulation of the receiver. We could not find any significant differences in the predictive control phase of the handover phase. However, the data reveals that the force overshoot of the receiver at the time when the passer fully releases the object is reduced in the glove condition. This effect most likely arises from the interaction between the partners in the feedback control phase of the handover phase due to its late occurrence. Due to the fact that the experimenter took the role of the receiver over the whole experiment, the power of the study design to answer this question was suboptimal. To test for this effect adequately, subject receiver variance has to be introduced into the study design together with conditions where the sensory status of the passer is unclear to have a more optimal reference. Furthermore, since the receiver had to stick to a preset protocol of conditions and had to concentrate on producing the correct reaching velocities, attentional shifts could be an explanation why we did not find such effects in this current study.

In the present study, motion capture data was primarily used to control experimental manipulation like the reaching velocity of the receiver. However, interesting exploratory effects could be observed in the vertical object movement during the handover phase. More specifically, in all conditions where visual information of the passer was restricted, the vertical object displacement was significantly larger compared to conditions where vision was accessible. One assumption can be that the vertical object displacement is an amplified cue for the sensory restricted passer that the receiver has a stable grasp of the object by unloading the passer from the load force. From a biomechanical perspective, this cue is highly reliable because when the passer is holding the object in place, the only way to accelerate the object is by an additional force that has to come from the receiver. Furthermore, since the vertical object displacement can be sensed by haptic sensors in the skin, muscles, and joints, the information is also available in all blind conditions. This assumption could be tested in a future experiment where the vertical movement of the object is systematically restricted during the handover.

## Methods

### Participants

Twenty-two healthy and right-handed volunteers were involved in the study (six males; age: 23.4 ± 2.4 years). All participants had normal or corrected-to-normal vision and reported no history of injuries in the upper limbs. All participants gave informed consent of their participation in the experiment. Each person received course credit for their participation (approximately 40 min total). The experiment was conducted in accordance with the ethical standards laid down in the Declaration of Helsinki. The protocol was approved by the Ethical Review Board of the Justus-Liebig-University, Giessen.

### Apparatus

**Figure 5:**
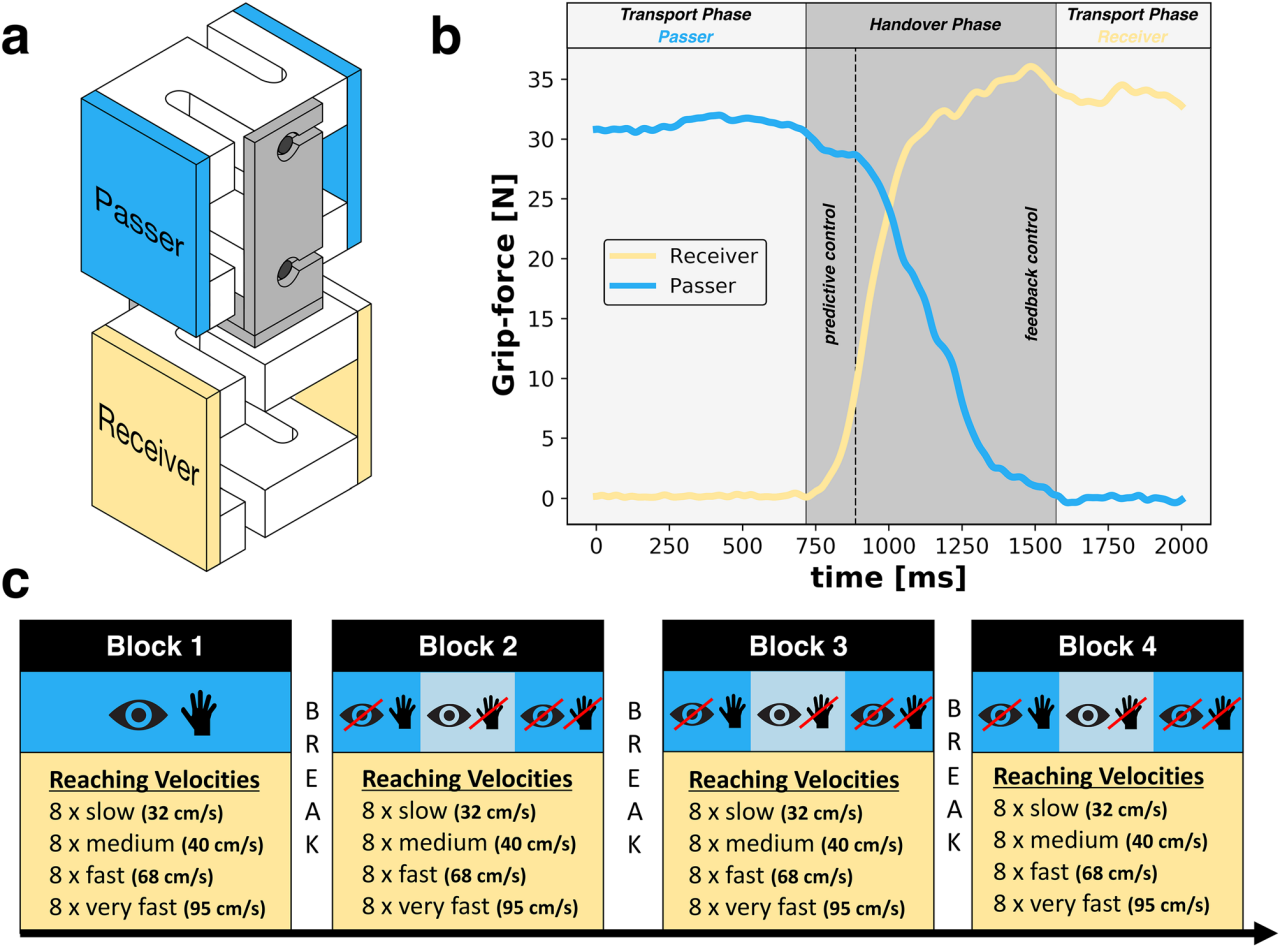
(**a**) Graphical illustration of the measuring device used to record the grip-forces of passer (blue) and (orange) receiver. Each side was constructed of two strain-gauge-sensors connected to a metal frame (grey). (**b**) Exemplary course of grip-forces of passer and receiver during one handover. (**c**) Schematic depiction of the study design. Each block consisted of 32 handovers with a 2-min break in between the blocks. Sensory information was manipulated on the passers side (shaded in blue) and reaching velocities were varied for the receiver (shaded in orange). In blocks 2–4, conditions were conducted counter balanced.

All data acquisitions were conducted using a custom-made measuring device (see Fig. 5a) (object dimensions: height = 14 cm, width = 5 cm, length = 9 cm). The device had two sides, an upper and a lower side, that enabled simultaneous measurements of grip-forces on each side. Forces at each side were measured by two strain-gauge-sensors whose signals were summed up in order to yield total grip-forces (GF). The total weight of the measuring device was 1.8 kg. Because of the weight of the object, participants were forced to use a power grip (instead of a pinch grip) to hold and grasp the object. Before each data acquisition, the force sensors were individually calibrated without a load and using normed weights of 2.5, 5.0, 7.5, and 10.0 kg. After the calibration, the object was reassembled and tested with a baseline measurement (static, no force applied to sensors). While passing the object to the receiver, both persons sat at a table with the following dimensions: height = 74 cm, length and width = 70 cm. Force data was recorded with a sampling rate of 1000 Hz.

### Study design

In order to meet the aims of the study, two independent variables have been included. First, the sensory information available to the passer and, second, the reaching velocity of the receiver in the first phase of the handover (i.e. transport phase passer; see also^5^). In detail, the passer’s visual and tactile information were manipulated. Vision was restricted by covering the passers eyes so that visual information could not be used in all three phases of the handover. Tactile signals were manipulated by letting the passer wear thick gloves. Altogether, handovers were recorded without any manipulation, with only one manipulation (i.e. ether visual or tactile), and with both manipulations applied simultaneously.

For the variation of reaching velocities, the receiver was asked to approach the object with four different velocities, i.e. slow (32 cm/s), medium (40 cm/s), fast (68 cm/s), and very fast (95 cm/s). Throughout the whole study, the experimenter took the role of the receiver. In preparation of the experiment, the experimenter was trained to reliably produce the asked reaching velocities but was not informed further about specific hypotheses of the study. At the beginning of each trial, the receiver was informed about the reaching velocity that had to be produced on a computer screen, which was placed diagonally behind the passer. To control the actual produced velocity, six optoelectric Vicon cameras with reflective markers were used to capture the position of the receiver’s hand (acquisition frequency 200 Hz). Additional markers were attached to the handover object to quantify the movement of the object itself.

In all trials, grip-force profiles of passer and receiver were recorded simultaneously. In total, the participants had to execute 128 handovers divided into four blocks (32 trials each block) with 2-min breaks in between. In the first block, participants executed handovers without any manipulations. From the second block, conditions were recorded in a randomized order. For each combination of sensory information (passer) and reaching velocity (receiver), we recorded eight trials (see Fig. 5c).

### Raw data processing and calculation of parameters

All data processing was conducted using Python 3.7 64 bit. First, the raw data of the force sensors was filtered with a fourth order low pass Butterworth filter with a cut-off frequency of 20 Hz. Calibration matrices (one for each sensor) were used to calculate a force signal in Newton. Afterwards, the measured forces of the top two sensors were summed to yield total grip-force of the passer. The same was done for the two sensors on the bottom of the object to yield the receiver’s total grip-force. Baseline correction was conducted separately for passer and receiver data. To correct the baseline of the passer’s and receiver’s force profile, the average force in a 50 ms time window (i.e., 50 data frames) in which the passer had no contact to the object was calculated. The kinematic data was filtered with a fourth order low pass Butterworth filter with a cut-off frequency of 10 Hz. To match the rate of the force data (1000 Hz) the kinematic data (200 Hz) was interpolated. After raw force data has been processed, all recordings were synchronized to the moment when the two force profiles intersect during handover phase. In order to discard irrelevant time periods, we cut the data, so that 1500 data frames before and a maximum of 1500 data frames after the intersection were left.

In order to detect the beginning of the passer’s release, zero-crossings in the force rate profile were used to find the last point before the rate was only decreasing (e.g. the passer was continuously releasing the object) until the object was fully released. To detect the end of the passer’s object release, a threshold algorithm was used with two conditions. The force rate had to be below 1 N/s and the value, which was found had to be below 0.5 N. The second condition was necessary because in some trials the object was not completely still at the end of the handover. Analogously, first contact of the receiver with the object was determined using the force rates of the receiver. The first contact of the receiver was subsequently used to re-synchronize and cut the data. Using these parameters, the *handover duration* was defined as the time interval between the first contact of the receiver and the last contact of the passer with the object. The time interval between the receiver’s initial contact with the object and the passers start of force release was defined as a measure for how reactive/anticipative the passer’s force release is (i.e. *Onset-Delay)*.

Furthermore, the reaching velocity was calculated from the kinematic data for each trial to reassure that it was conform with the target reaching velocity. If that was not the case, the actual velocity was used to relabel the trial. 11.9% of the trials were relabelled to an adjacent category (e.g. slow to medium). Relabelling to a distant category did not occur.

In all figures with confidence bands the point-based-resampling-technique (PBRT^16^) was used, which leads to 95% confidence bands that make it possible to reliably evaluate statistical significance.

### Expectations and statistical analysis

In the statistical analysis, data was used to get answers to three questions.

- How does the passer modulate his/her grip-force behaviour when having restricted sensory information about the receiver’s reaching movement and the interaction itself?
- How does the passer behave when the receiver approaches the object with different reaching velocities?

Regarding (1), it was assumed that the passer’s increased uncertainty about the receiver’s state is compensated by a more reactive release of the object. Specifically, a larger time difference between the receiver’s initial contact and the start of the passer’s force release (i.e. *onset difference*) was expected. An increased uncertainty about the receiver’s state could also lead to a larger grip-force of the passer in general. According to the findings of Controzzi et al.^8^, the passer releases the object with a higher rate of grip-force reduction in trials with sensory manipulation compared to trials where visual and tactile information are not manipulated.

For question (2), the passer will start releasing the object earlier relative to the receiver’s initial contact with the object. This would show in a shorter onset difference in trials with fast reaching velocities compared to trials with slow or medium reaching velocities of the receiver. Furthermore, it was assumed that the receiver will have a higher rate of grip-force production in trials with fast reaching velocities. If the passer uses the reaching velocity of the receiver as a predictor for the rate of grip-force production, the passer should react with an increased rate of grip-force reduction.

With respect to question (3), the receiver was expected to behave differently when passer has impaired sensory information. If the receiver anticipates a higher onset difference, the receiver should react with a smaller force production rate so that the total amount of grip-forces applied to the object gets minimized.

For statistical testing, repeated measurement ANOVAs were calculated including both factors (sensory and reaching velocity variations). ANOVAs were calculated for six predefined variables (i.e. handover duration, grip-force of passer in transport phase, grip-force of receiver when passer fully released the object, force rates of passer and receiver during handover phase, onset-delay, and the receiver’s force rate in the first 200 ms). Normality of error residuals was checked using Q–Q-plots in JASP (version 0.11.1). Sphericity was tested using Mauchly’s test assuming a violation of sphericity when *p* < 0.05. In case of sphericity violations, Greenhouse–Geisser corrections were applied. For post hoc comparisons, the Holm algorithm was used to correct for multiple comparisons. The alpha level for all statistical tests was set to *α* = 0.05. All *p*-values are the result of two-sided testing. To assure validity of the statistical results, the Bayesian equivalent to the repeated measures ANOVA was computed to yield Bayes Factors (*BF*) that can be interpreted as the amount of evidence for the null-hypothesis before versus after seeing the data^17^. BFs were computed in JASP with default priors and interpreted according to Raftery^18^.

## Data Availability

The datasets analysed during the current study are available from the corresponding author on reasonable request.
